# Clonal Expansion of Biofilm-Forming *Salmonella* Typhimurium ST34 with Multidrug-Resistance Phenotype in the Southern Coastal Region of China

**DOI:** 10.3389/fmicb.2017.02090

**Published:** 2017-10-27

**Authors:** Wanli Li, Yinghui Li, Yao Liu, Xiaolu Shi, Min Jiang, Yiman Lin, Yaqun Qiu, Qian Zhang, Qiongcheng Chen, Li Zhou, Qun Sun, Qinghua Hu

**Affiliations:** ^1^Key Laboratory of Bio-resources and Eco-environment of the Ministry of Education, College of Life Sciences, Sichuan University, Chengdu, China; ^2^Shenzhen Major Infectious Disease Control Key Laboratory, Shenzhen Center for Disease Control and Prevention, Shenzhen, China; ^3^School of Life Sciences, Shenzhen University, Shenzhen, China

**Keywords:** *Salmonella* Typhimurium, antibiotic resistance, biofilm, genetic diversity, ST34

## Abstract

To disclose the antibiotics susceptibility and wide adaptability of commonly occurring genotypes of *Salmonella* Typhimurium, the antibiotic resistance and biofilm formation of different multi-locus sequence typing (MLST) types of a collection of 240 *S*. Typhimurium isolates (33 food and 207 clinical ones) during 2010–2014 in Shenzhen were analyzed. Among these strains, 167 was ST34 (69.58%), and 57 was ST19 (23.75%), respectively. A total of 159 (95.21%) ST34 strains displayed the multidrug resistant phenotype (≥ three classes of antibiotic), whereas only 23 (40.35%) ST19 ones did (*P* < 0.01). Moreover, a relative high proportion (72.46%) of ST34 isolates was classified as moderate to strong biofilm-producers, while only 15.79% of ST19 (*P* < 0.01) was. Among the food isolates, more than half (51.52%) were from livestock products, among which 41.18% classified as moderate to strong biofilm-producers. In summary, this study highlights the expansion of *S*. Typhimurium ST34 of strong biofilm-forming ability and multidrug resistance in the southern coastal region of China. Therefore, monitoring the occurrence of ST34 *S*. Typhimurium in food sources, especially in livestock products, and taking appropriate measures to control *Salmonella* spp. infections via decreasing biofilm formation should be addressed.

## Introduction

The genus *Salmonella* is a major causative agent of foodborne illnesses, responsible for more than 90 million cases of salmonellosis annually across the globe (Majowicz et al., 2010; Havelaar et al., 2015). In China, *Salmonella* Typhimurium was reported as the most frequently isolated serotype of non-typhoidal *Salmonella* from foodborne illnesses (Liang et al., 2015). The fast growing multidrug resistance associated with extensive antibiotic usage is creating new challenges for the control and prevention of *Salmonella* spp. infections worldwide (Hazards, 2009). In China, more than 75% of *Salmonella* spp. isolates studied are resistant to at least one antibiotic (Wang et al., 2013; Zhang et al., 2013). The mechanism of the drug resistance in *Salmonella* is complex, including its resistance at the cellular level and the adaptive resistance (Penesyan et al., 2015). Antibiotic resistance genes, one of resistance mechanisms at the cellular level, are frequently located on mobile genetic elements, such as plasmids, transposons, and integrons, which can transfer between foodborne bacteria and gut microbiota in human via bacteriophages (Annemieke et al., 2009; Muniesa et al., 2013).

In addition to the resistance at cellular level, antibiotic resistance also can be achieved by adaptive resistance, for example, producing biofilm (Corona and Martinez, 2013). The biofilm-forming ability can confer bacterial tolerance and thus protect them from suboptimal environments, antibiotics, and disinfectants, consequently extremely difficult to be eradicated (Nguyen and Yuk, 2013; Vázquez-Sánchez et al., 2014). It has been reported that the ability to form biofilms by foodborne pathogens was closely related to human illness and the increased risk of food safety (Steenackers et al., 2012). *Salmonella* spp. have demonstrated the ability to form biofilms on abiotic surfaces, plant surfaces, and animal epithelial cells in several studies that were focused on characterizing biofilm-forming ability in *S*. Typhimurium clinical isolates (Steenackers et al., 2012). However, the number of *S*. Typhimurium isolates analyzed in these studies is relatively small. For example, Papavasileiou et al. reported that 17 out of 27 *S*. Typhimurium strains isolated from children with gastroenteritis formed biofilms (Papavasileiou et al., 2010). O'Leary et al. reported that 22 clinical isolates out of 30 were moderate biofilm-producers and the rest as weak ones (O'Leary et al., 2013). In China, there is a paucity of published literature on the biofilm-forming ability of clinical *S*. Typhimurium isolates. Therefore, investigating the biofilm forming ability of *S*. Typhimurium isolates obtained from food and clinical sources will help us in understanding their adaptability, so as to identify the potential risk of *Salmonella* infection.

Multi-locus sequence typing (MLST) is a genotyping method that can reveal the genetic relatedness of bacterial strains and thus facilitate tracking their evolutionary paths. According to the MLST database (http://mlst.warwick.ac.uk/mlst/dbs/Senterica), a total of 63 sequence types (STs) were found among the global *S*. Typhimurium serotype, with ST34 (57%) and ST19 (28.4%) being the most common STs in Asia. For the antibiotic resistance phenotypes, Zankari et al. observed that ST34 isolates were more resistant than ST19 ones in Denmark (Zankari et al., 2012). In China, there is still few published literature on antibiotic resistance for different STs. Moreover, biofilm formation, another phenotypic characteristic closely related to drug resistance, has been still poorly investigated for different STs.

In this study, the antibiotic resistance and biofilm formation phenotypes for different MLST types were investigated in 240 *S*. Typhimurium isolates collected during 2010–2014, including 207 clinical isolates and 33 food ones, to understand the adaptability of the most common genotypes, so as to provide measures for controlling the prevalence of *S*. Typhimurium.

## Materials and methods

### Bacterial strains

A collection of 27,990 stool samples from outpatients with acute diarrhea was obtained from 11 sentinel hospitals in Shenzhen, China between 2010 and 2014. From this collection, a total of 393 *S*. Typhimurium clinical isolates were identified by using standard biochemical and serological tests. Isolates obtained within 2 months with the same antibiotic susceptibility and PFGE pattern were confirmed as outbreak isolates by epidemiological investigation. The outbreak isolates were excluded for this study. So based on the PFGE and epidemiological data of the 393 *S*. Typhimurium isolates, 207 strains with 168 different PFGE patterns and other 33 food isolates recovered from product of poultry or livestock, ready to eat food, and aquatic products (Table S1) were selected for this study, The stock cultures were maintained at −80°C in tryptone soy broth (TSB; Oxoid Ltd., Cambridge, U.K.) supplemented with 20% glycerol.

### MLST

Amplification of the internal fragments of seven housekeeping genes was performed according to the MLST scheme at http://mlst.ucc.ie/mlst/dbs/Senterica. Sequencing of all PCR products was conducted using the respective primer pair (Table 1) used for PCR amplification (Invitrogen, China). The eBURST v3.0 was used to identify clonal complexes (CCs) in relation to the strains in the *Salmonella enterica* MLST database.

**Table 1:** PCR and sequencing primer for MLST used in this study.

**Primer designation**	**Sequence (5′-3′)**
thrA-F	GTCACGGTGATCGATCCGGT
thrA-R	CACGATATTGATATTAGCCCG
purE-F	GACACCTCAAAAGCAGCGT
purE-R	AGACGGCGATACCCAGCGG
sucA-F	CGCGCTCAAACAGACCTAC
sucA-R	GACGTGGAAAATCGGCGCC
hisD-F	GAAACGTTCCATTCCGCGC
hisD-R	GCGGATTCCGGCGACCAG
aroC-F	CCTGGCACCTCGCGCTATAC
aroC-R	CCACACACGGATCGTGGCG
hemD-F	GAAGCGTTAGTGAGCCGTCTGCG
hemD-R	ATCAGCGACCTTAATATCTTGCCA
dnaN-F	ATGAAATTTACCGTTGAACGTGA
dnaN-R	AATTTCTCATTCGAGAGGATTGC

### Antimicrobial susceptibility tests

Antimicrobial susceptibility testing of all *S*. Typhimurium isolates was performed using the disk-diffusion method as described by the Clinical and Laboratory Standards Institute (CLSI, 2012). Antibiotic discs were purchased from Oxoid (Oxoid Ltd, England). A panel of 18 antimicrobial agents covering seven CLSI classes of antibiotics was used along with the zone diameter to determine if the isolates were resistant: ampicillin (AMP; 10 μg, ≦ 13 mm), amoxicillin/clavulanic acid (AMC; 20/10 μg, ≦ 13 mm), cephalothin (CEP; 30 μg, ≦ 14 mm), ceftazidime (CAZ; 30 μg, ≦ 17 mm), ceftriaxone (CRO; 30 μg, ≦ 19 mm), cefepime (FEP; 30 μg, ≦ 14 mm), cefoxitin (FOX; 30 μg, ≦ 14 mm), amikacin (AMK; 30 μg, ≦ 14 mm), gentamicin (GEN; 10 μg, ≦ 12 mm), kanamycin (KAN; 30 μg, ≦ 13 mm), streptomycin (STR; 10 μg, ≦ 11 mm), nalidixic acid (NAL; 30 μg, ≦ 13 mm), ciprofloxacin (CIP; 5 μg, ≦ 20 mm), levofloxacin (LVX; 5 μg, ≦ 13 mm), trimethoprim/sulfamethoxazole (SXT; 1.25/23.75 μg, ≦ 10 mm), trimethoprim (TMP; 5 μg, ≦ 10 mm), chloramphenicol (CHL; 30 μg, ≦ 12 mm), and tetracycline (TCY; 30 μg, ≦ 11 mm). Breakpoints for sensitive, intermediate and resistant were defined by the CLSI (2012). The *Escherichia coli* strain ATCC 25922 was used as a quality control.

### Biofilm production

Biofilm production was assessed using a previously described protocol with some modifications (Ben-Jacob et al., 2013). Briefly, overnight bacterial cultures were grown at 37°C with shaking, and were adjusted to give an optical density (OD_600_) of 0.01 by diluted (1:10) TSB. A 100 μL volume of bacterial suspension was added to each well of a 96-well plate and incubated at 28°C for 48 h without shaking. The medium was then discarded, and each well was washed with water and stained with 100 μL of crystal violet for 15 min. Finally, the wells were washed three times with water and biofilm formation was assayed by the addition of 100 μL of 95% ethanol in order to measure the OD at 570 nm. Each isolate was assayed in eight individual replicates. The negative controls contained only 100 μL of diluted TSB.

Based on the data obtained in the present investigation and the criteria suggested (Díez-García et al., 2012), strains were classified into one of four categories: non-biofilm producer, weak biofilm producer, moderate biofilm producer, or strong biofilm producer.

### Statistical analysis

All statistical analyses were performed using the SPSS 13.0 for Windows software; a *P* < 0.05 was considered to be significant. Differences in the biofilm category were determined by analysis of variance (ANOVA).

## Results

### MLST analysis

In order to understand the distribution and the evolution of the clones, MLST was used to genetically type the isolates collected. Irrespective of their collecting sources, a total of 167 (167/240; 69.58%) isolates belong to genotype ST34, and 57 (57/240; 23.75%) isolates were ST19 (Table 2). Additionally, three major MLST types (ST34, ST19, and ST36) were found in both the clinical and food isolates (Figure S1). For the food isolates, five different MLST types were observed, with ST34 (18/33; 54.55%) being the most common MLST type, followed by ST19 (11/33; 33.33%). Among five different MLST types, only ST34 and ST19 belong to complex one (Table 2, Figure S1A). For the clinical isolates, a total of eight different MLST types were observed in *S*. Typhimurium strains, with ST34 (71.98%; 149/207) being the most prevalent MLST type, followed by ST19 (22.22%; 46/207). And seven out of eight STs belong to a single clonal complex (complex one) with ST19 as the founder (Table 2, Figure S1B). In addition, a new ST, i.e., ST1963, was identified from clinical isolates.

**Table 2:** MLST sequence types (STs) of 240 *S*. Typhimurium isolates.

**STs**	**ST Complex**	**Source**	**Total number of isolates**
ST34	1	Food (18); clinical (149)	167
ST19	1	Food (11); clinical (46)	57
ST36	138	Food (2); clinical (4)	6
ST1544	1	Clinical	4
ST99	1	Clinical	1
ST1557	1	Clinical	1
ST1958	1	Clinical	1
ST516	67	Food	1
ST241	33	Food	1
ST1963^a^	1	Clinical	1

a *ST1963: a new ST was identified*.

### Distribution of MDR

Antimicrobial susceptibility testing was performed on 18 different antibiotics, which were divided into seven antibiotic classes, including penicillins, cephems, aminoglycosides, folate pathway inhibitors, quinolones, tetracyclines and phenicols, to evaluate the antimicrobial resistance profile of the *S*. Typhimurium isolates recovered from food and clinical sources. Irrespective of their source isolated, a total of 191 (191/240; 79.58%) isolates were resistant to at least three classes of antibiotics and 33 (33/240; 13.75%) isolates were resistant to seven classes of antibiotics (Table 3). A total of 81.64% (169/207) clinical isolates were resistant to at least three classes of antimicrobial agents, which is severer than food isolates (66.67%; 22/33).

**Table 3:** Resistance to multiple antibiotic classes of the 240 *S*. Typhimurium isolates.

**Number of antibiotic classes to be resistant^a^**	**Number of isolates from the sources of**		**Total**
	**Food (%)**	**Clinical (%)**	
0	0 (0.00)	5 (2.42)	5 (2.08)
1	4 (12.12)	11 (5.31)	15 (6.25)
2	7 (21.21)	22 (10.63)	29 (12.08)
≥ 3	22 (66.67)	169 (81.64)	191 (79.58)
3	6 (18.18)	13 (6.28)	19 (7.92)
4	4 (12.12)	25 (12.08)	29 (12.08)
5	9 (27.27)	29 (14.01)	38 (15.83)
6	3 (9.09)	69 (33.33)	72 (30.00)
7	0 (0.00)	33 (15.94)	33 (13.75)
Total	33 (100.00)	207 (100.00)	240 (100.00)

a *Eighteen antibiotics were divided into seven classes: penicillins, cephems, aminoglycosides, folate pathway inhibitors, quinolones, tetracyclines, and phenicols*.

### Biofilm formation

Biofilm-forming ability of the *S*. Typhimurium isolates was analyzed to investigate the adaptability to the suboptimal environments of these strains (Table 4). Irrespective of their source of isolation, a total of 188 (188/240; 78.33%) isolates were categorized as biofilm producer, among which 132 (132/188; 70.21%) isolates were moderate or strong biofilm producers. The food isolates are mainly (17/33; 51.52%) obtained from livestock products, with 41.18% (7/17) strains classified as moderate or strong biofilm producers (Table 4). Additionally, 11 (11/33; 33.33%) isolates were obtained from poultry products, in which two (2/11; 18.18%) strains were classified as moderate or strong biofilm producer. Then chi-square test was used to analyze the relationship between the collecting sources (livestock, poultry, aquatic and ready-to-eat) and the frequency of biofilm formation in food isolates, whereas there was no significant correlation between the collecting sources and the frequency of biofilm formation (*P* > 0.05). Among the 176 clinical strains with ability to form biofilms, 120 (68.18%; 120/176) were classified as moderate to strong biofilm producers. The rate of biofilm formation for *S*. Typhimurium strains isolated from food sources (12/33; 36.36%) was lower than that of the clinical ones (176/207; 85.02%).

**Table 4:** Biofilm formation of the total 240 *S*. Typhimurium isolates.

**Biofilm formation category^a^**	**Food source (%)**				**Clinical source (%)**	**Total number of isolates (%)**
	**Livestock**	**Poultry**	**Aquatic**	**Ready-to-eat**		
N	10 (30.30)	9 (27.27)	0 (0.00)	2 (6.06)	31 (14.98)	52 (21.67)
W	0 (0.00)	0 (0.00)	0 (0.00)	0 (0.00)	56 (27.05)	
M	4 (12.12)	0 (0.00)	1 (3.03)	0 (0.00)	39 (18.84)	188 (78.33)
S	3 (9.09)	2 (6.06)	1 (3.03)	1 (3.03)	81 (39.13)	
Total number of isolates	33				207	240

a *Different categories of biofilm production are represented by the following abbreviations: N, not biofilm producer; W, weak; M, moderate; S, strong*.

### Antibiotic resistance and biofilm formation for different MLST types

The majority (159/167; 95.21%) of the ST34 isolates were phenotypically resistant to at least three classes of antimicrobial agents, while only 40.35% (23/57) of the ST19 isolates were. Therefore, ST34 isolates tended to be resistant to more antibiotic classes than ST19 ones (chi-square test; *P* < 0.01). It is mentionable that all ST36 isolates (*n* = 6) were resistant to three or more classes of antimicrobial agents.

The analysis of biofilm formation for each MLST type in *S*. Typhimurium strains was performed to understand their adaptability in different environments. A tendency was observed among the ST34 and ST19 isolates (Table 5), where only 8.98% (15/167) ST34 isolates, compared with 50.88% (29/57) ST19, were classified as non-biofilm producer. Moreover, a high proportion of ST34 isolates (121/167; 72.46%) were classified as moderate to strong biofilm producers, while in contrast, only 15.79% (9/57) ST19 isolates were so, indicating that ST34 strains were more likely to be moderate or strong biofilm producers than ST19 strains (chi-square test; *P* < 0.01).

**Table 5:** Antibiotic resistance and biofilm formation of 240 *S*. Typhimurium isolates of different MLST types.

**MLST type^a^**	**No. (%) of antibiotic classes to which isolates were resistant^b^**									**Biofilm formation category^c^**				**Total**
	**0**	**1**	**2**	**≥ 3**	**3**	**4**	**5**	**6**	**7**	**N**	**W**	**M**	**S**	
ST34	1 (0.60)	2 (1.20)	5 (2.99)	159 (95.21)	14	24	33	62	26	15 (8.98)	31 (18.56)	37 (22.16)	84 (50.30)	167
ST19	4 (7.02)	10 (17.54)	20 (35.09)	23 (40.35)	1	4	4	7	7	29(50.88)	19 (33.33)	6 (10.53)	3 (5.26)	57
ST36	0 (0.00)	0 (0.00)	0 (0.00)	6 (100.00)	4	1	0	1	0	3 (50.00)	3 (50.00)	0 (0.00)	0 (0.00)	6
ST1544	0 (0.00)	1 (25.00)	3 (75.00)	0 (0.00)	0	0	0	0	0	2 (50.00)	1 (25.00)	1 (25.00)	0 (0.00)	4
ST1557	0 (0.00)	1 (100.00)	0 (0.00)	0 (0.00)	0	0	0	0	0	1 (100.00)	0 (0.00)	0 (0.00)	0 (0.00)	1
ST1963	0 (0.00)	0 (0.00)	0 (0.00)	1 (100.00)	0	0	0	1	0	0 (0.00)	0 (0.00)	0 (0.00)	1 (100.00)	1
ST1958	0 (0.00)	0 (0.00)	0 (0.00)	1 (100.00)	0	0	0	1	0	0 (0.00)	1 (100.00)	0 (0.00)	0 (0.00)	1
ST516	0 (0.00)	0 (0.00)	1 (100.00)	0 (0.00)	0	0	0	0	0	1 (100.00)	0 (0.00)	0 (0.00)	0 (0.00)	1
ST241	0 (0.00)	0 (0.00)	0 (0.00)	1 (100.00)	0	0	1	0	0	1 (100.00)	0 (0.00)	0 (0.00)	0 (0.00)	1
ST99	0 (0.00)	1 (100.00)	0 (0.00)	0 (0.00)	0	0	0	0	0	0 (0.00)	1 (100.00)	0 (0.00)	0 (0.00)	1

a *Data in the table are expressed as the number of isolates*.

b *Eighteen different antibiotics were divided into seven classes of antibiotics: penicillins, cephems, aminoglycosides, folate pathway inhibitors, quinolones, tetracyclines and phenicols*.

c *Different categories of biofilm production are represented by the following abbreviations: N, not biofilm producer; W, weak biofilm producer; M, moderate biofilm producer; S, strong biofilm producer*.

## Discussions

Non-typhoidal *Salmonella enterica* are one of the leading causes of food-borne diseases (Majowicz et al., 2010). We found that *S*. Typhimurium (29.44%; 393/1335) was the most frequently isolated serotype among 1,335 non-typhoidal *Salmonella* isolates obtained from diarrheal disease surveillance between 2010 and 2014 (data not shown) in Shenzhen, China.

In order to evaluate the antimicrobial resistance profile of the *S*. Typhimurium isolates recovered from foods and clinical sources, antimicrobial susceptibility testing was performed by using 18 different antibiotics. For isolates recovered from food sources, no isolate was resistant to the cephem class (CEP, CAZ, CRO, FEP, and FOX; Table S2), while a relatively high number of isolates were resistant to NAL (28/33; 84.8%), STR (25/33; 75.8%), and TCY (21/33; 63.6%). For clinical isolates, the *S*. Typhimurium strains were the most commonly resistant to CIP (180/207; 87.0%), AMP (155/207; 74.9%), and AMC (108/207; 52.2%). The antibiotic resistance rates of CAZ and CRO increased in 2014, reaching 23.5% and 21.0%, respectively (Table S3). Although the use of antimicrobials is not generally recommended to treat salmonellosis, fluoroquinolones and extended-spectrum β-lactams have been commonly applied to treat salmonellosis infections in China. The prevalence of resistance to CIP, AMP and AMC has been maintained at a higher level among *S*. Typhimurium clinical isolates from China, in comparison to those from other countries (Campioni et al., 2012; Pérez-Moreno et al., 2013; Abraham et al., 2014).

Multi-locus sequence typing (MLST) provides a basis for typing and understanding the evolution of *S*. Typhimurium clones (Lan et al., 2009). In this study, ST34 was the most common MLST type, followed by ST19. ST34 differs from ST19 by only one allele (*dnaN*), in which a single base change occurs. Although a total of 10 MLST types were observed in 240 isolates from food and clinical sources, seven out of which belong to a single clonal complex (complex one) with ST19 as the founder. These findings indicated that 96.67% (232/240) *S*. Typhimurium strains in Shenzhen, China were classified as complex one.

Among 10 different STs, seven STs belong to a single clonal complex, in which the phenotypic characteristics for each MLST type were markedly different. We found that ST34 isolates were resistant to more antibiotic classes than ST19 isolates. This is in agreement with the results seen in previous studies (Zankari et al., 2012; Wong et al., 2013).

Biofilms have become a focus in food safety problems (Steenackers et al., 2012). In this study, a preliminary correlation analysis revealed a significant positive association between the ability to form biofilms and the number of antibiotic classes to which a strain was resistant (*P* ≤ 0.001; Figure S2). Therefore, it is possible that biofilm formation will confer upon bacteria improved adaptation to an unfavorable environment, thereby promoting the evolution of the multi-drug resistant phenotype. It is mentionable that the majority (72.46%; 121/167) of ST34 isolates were classified as moderate or strong biofilm producers in the present study. Additionally, all 12 isolates recovered from food sources, which were classified as moderate or strong biofilm producers, were ST34 genotype. These phenotypes of ST34 might explain why ST34 *S*. Typhimurium was the most frequently isolated genotype from diarrheal outpatients in China.

In China, studies on the comparative biofilm-forming ability of *S*. Typhimurium from diverse food sources are very limited. It has been reported that five out of 23 *Salmonella* spp. strains isolated from a chicken slaughter plant in China were biofilm-producers, and this can help in the establishment of appropriate regulatory measures for farming practice (Wang et al., 2013). In the present study, although no significant correlation was observed between the collecting sources and the frequency of biofilm formation (*P* > 0.05) in food isolates, strains isolated from livestock products (7/17; 41.18%) were more likely to be classified as biofilm-producer than those from poultry (2/11; 18.18%). As previously reported, a major source of infection of *S*. Typhimurium in humans was the consumption of livestock products (Sallam et al., 2014; Doménech et al., 2015), which is consistent with our investigation here (Table 4). These findings may assist optimizing Hazard Analysis and Critical Control Point (HACCP) strategies in livestock abattoirs.

## Conclusion

In summary, this study demonstrated the phenotype of antibiotic resistance and biofilm formation for different MLST types of *S*. Typhimurium isolates from various food sources and acute diarrheal outpatients in Shenzhen, China. ST34 was detected as the most common genotype and was associated with multiple antibiotic resistance and strong ability to form biofilm. These findings may help our understanding the expansion of *S*. Typhimurium in southern coastal region of China. In addition, based on the analysis of biofilm formation for different MLST types of *S*. Typhimurium isolated from different sources, monitoring the occurrence of ST34 *S*. Typhimurium in various food sources, especially in the processing of livestock products, and taking appropriate measures to tackle biofilm formation should be addressed.

## Author contributions

All authors contributed substantially to the work reported. WL, QS, and QH designed experimental strategies. WL, YhL, QC, and QZ performed experiments and analyzed data. YhL, XS, MJ, YmL, YQ, and LZ performed collecting strains. WL, QS, and QH wrote the manuscript.

### Conflict of interest statement

The authors declare that the research was conducted in the absence of any commercial or financial relationships that could be construed as a potential conflict of interest.
