# Supplementary material for: Clonal Expansion of Biofilm-Forming Salmonella Typhimurium ST34 with Multidrug-Resistance Phenotype in the Southern Coastal Region of China
Source: Front Microbiol. 2017 Oct 27;8:2090. doi: 10.3389/fmicb.2017.02090 (PMC5674920; doi:10.3389/fmicb.2017.02090)
Supplement: Supplementary file 1 [file DataSheet1.DOC]

**Supplementary Materials for**

**Clonal expansion of biofilm-forming Salmonella Typhimurium ST34 with multidrug-resistance phenotype in the southern coastal region of China**

**This file includes:**

**Fig. S1**

**Fig. S2**

**Table S1**

**Table S2**

**Table S3**

**
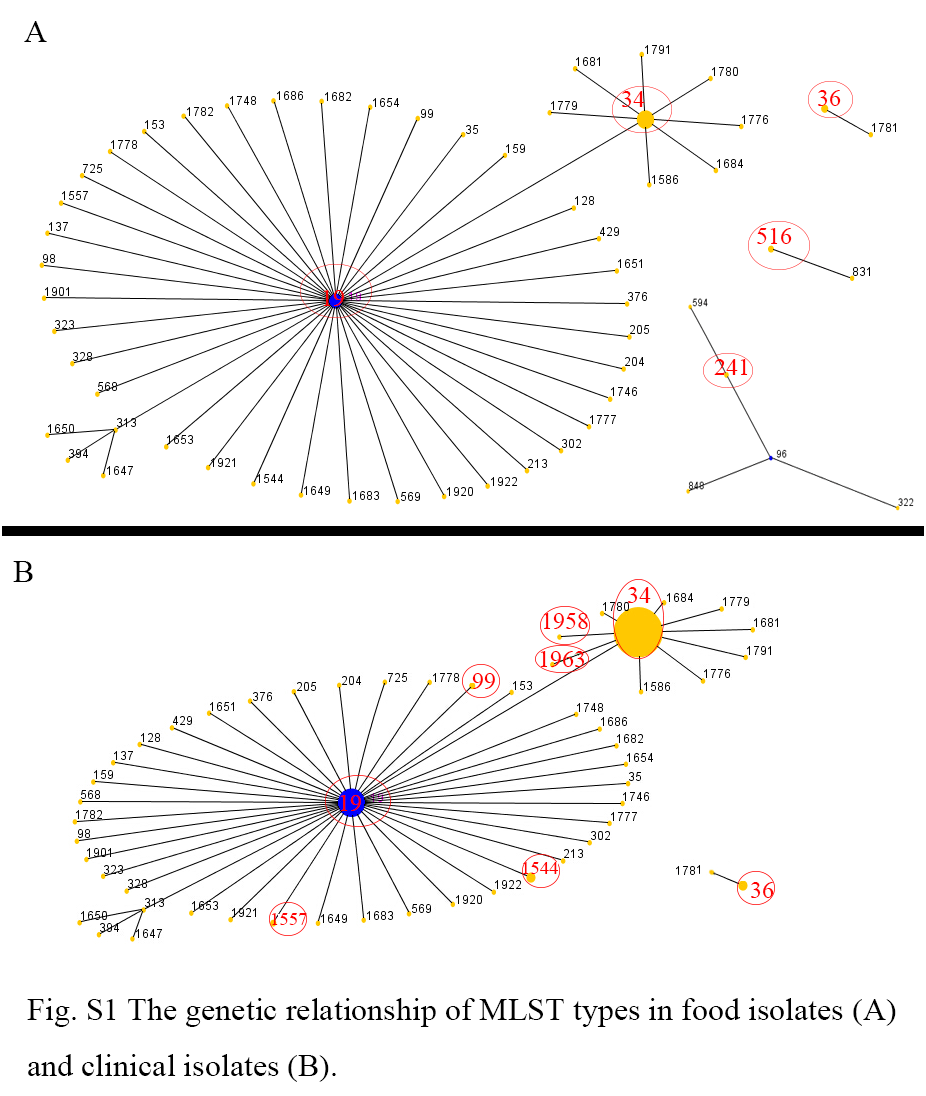
**

Fig. S1 The genetic relationship of MLST types in food (A) and clinical isolates (B).


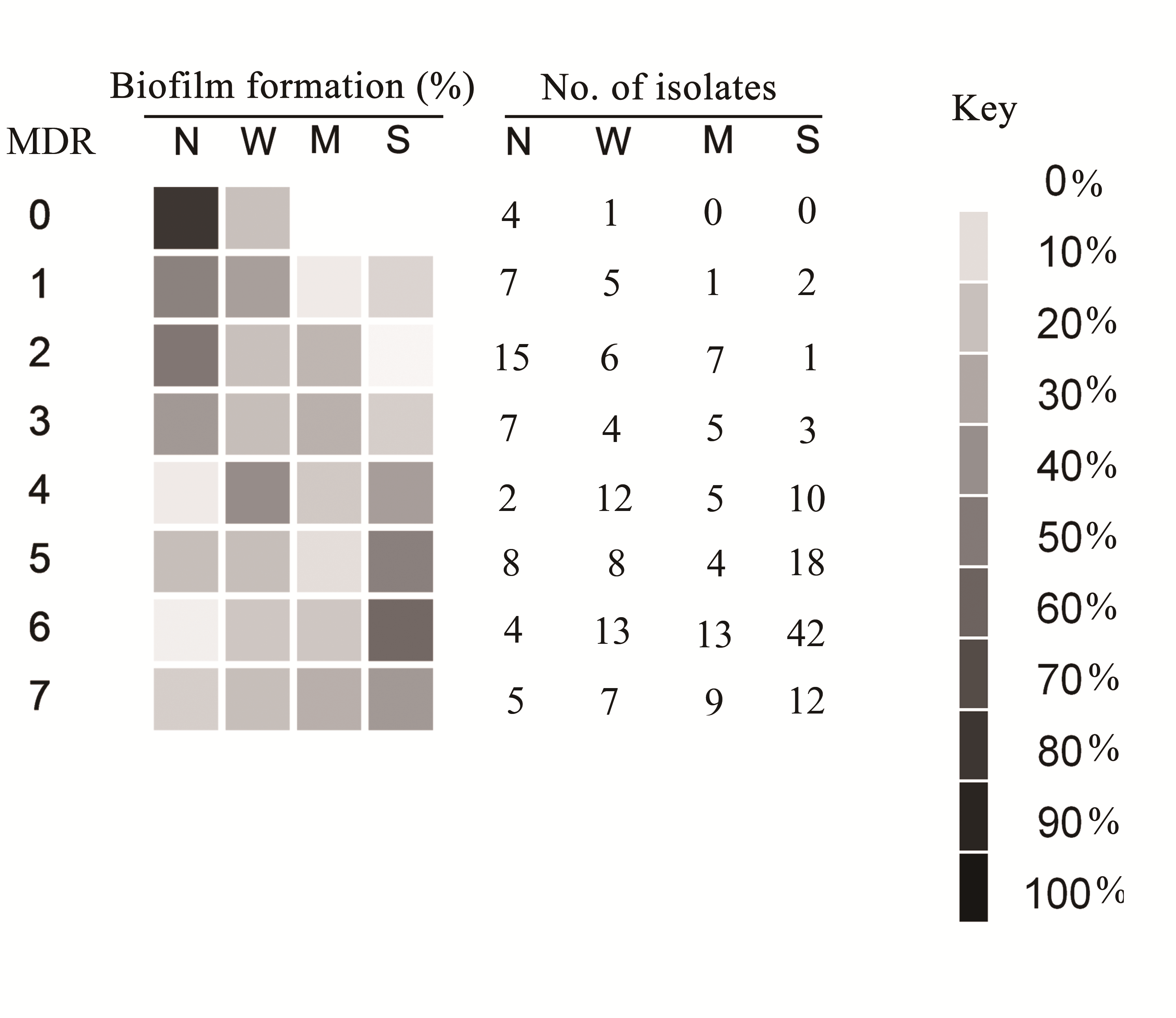


Fig. S2 The frequency of biofilm formation to different categories by the number of antibiotic-resistant classes (MDR) for 240 isolates. Eighteen antibiotics were divided into seven classes of antibiotics: penicillins, cephems, aminoglycosides, folate pathway inhibitors, quinolones, tetracyclines and phenicols. Different categories of biofilm production are represented by the following abbreviations: N, non-biofilm producer; W, weak biofilm producer; M, moderate biofilm producer; S, strong biofilm producer.

Table S1 PFGE patterns, biofilm formation and MLST types of 240 *S*. Typhimurium isolates.

| Strains | PFGE patterns | Source/type of sample | STs | Biofilm category ^a^ |
| --- | --- | --- | --- | --- |
| S10034 | JPXX01.SZ0096 | Patient/stool | ST19 | N |
| S10016 | JPXX01.SZ0021 | Patient/stool | ST19 | N |
| S13113 | JPXX01.SZ0025 | Patient/stool | ST34 | M |
| S13347 | JPXX01.SZ0078 | Patient/stool | ST34 | W |
| S13127 | JPXX01.SZ0006 | Patient/stool | ST34 | S |
| S13064 | JPXX01.SZ0059 | Patient/stool | ST34 | W |
| S13017 | JPXX01.SZ0063 | Patient/stool | ST34 | N |
| S13176 | JPXX01.SZ0055 | Patient/stool | ST34 | S |
| S13105 | JPXX01.SZ0132 | Patient/stool | ST34 | S |
| S13054 | JPXX01.SZ0119 | Patient/stool | ST34 | M |
| S13085 | JPXX01.SZ0276 | Patient/stool | ST34 | S |
| S13041 | JPXX01.SZ0085 | Patient/stool | ST34 | S |
| S13278 | JPXX01.SZ0131 | Patient/stool | ST34 | S |
| S12152 | JPXX01.SZ0103 | Patient/stool | ST34 | S |
| S11234 | JPXX01.SZ0282 | Patient/stool | ST34 | S |
| S11212 | JPXX01.SZ0002 | Patient/stool | ST34 | S |
| S11050 | JPXX01.SZ0013 | Patient/stool | ST34 | S |
| S11130 | JPXX01.SZ0123 | Patient/stool | ST34 | S |
| S11173 | JPXX01.SZ0047 | Patient/stool | ST34 | M |
| S10064 | JPXX01.SZ0079 | Patient/stool | ST34 | S |
| S10130 | JPXX01.SZ0078 | Patient/stool | ST34 | S |
| S10136 | JPXX01.SZ0078 | Patient/stool | ST34 | M |
| S10146 | JPXX01.SZ0215 | Patient/stool | ST34 | S |
| S10062 | JPXX01.SZ0010 | Patient/stool | ST34 | S |
| S14247 | JPXX01.SZ0226 | Patient/stool | ST34 | S |
| S14193 | JPXX01.SZ0221 | Patient/stool | ST34 | S |
| S14204 | JPXX01.SZ0006 | Patient/stool | ST34 | W |
| S14106 | JPXX01.SZ0200 | Patient/stool | ST34 | M |
| S14410 | JPXX01.SZ0258 | Patient/stool | ST34 | N |
| S14165 | JPXX01.SZ0272 | Patient/stool | ST34 | W |
| S14398 | JPXX01.SZ0054 | Patient/stool | ST34 | M |
| S14195 | JPXX01.SZ0281 | Patient/stool | ST34 | S |
| S14221 | JPXX01.SZ0192 | Patient/stool | ST34 | W |
| S14318 | JPXX01.SZ0006 | Patient/stool | ST34 | S |
| S14200 | JPXX01.SZ0191 | Patient/stool | ST34 | W |
| S14049 | JPXX01.SZ0203 | Patient/stool | ST1958 | W |
| S14379 | JPXX01.SZ0118 | Patient/stool | ST34 | S |
| S14120 | JPXX01.SZ0256 | Patient/stool | ST34 | M |
| S14392 | JPXX01.SZ0254 | Patient/stool | ST34 | M |
| S14378 | JPXX01.SZ0225 | Patient/stool | ST34 | M |
| S14232 | JPXX01.SZ0176 | Patient/stool | ST34 | N |
| S14313 | JPXX01.SZ0239 | Patient/stool | ST1544 | W |
| S14060 | JPXX01.SZ0157 | Patient/stool | ST19 | W |
| S14131 | JPXX01.SZ0247 | Patient/stool | ST1544 | M |
| S14084 | JPXX01.SZ0034 | Patient/stool | ST19 | W |
| S14196 | JPXX01.SZ0277 | Patient/stool | ST19 | W |
| S14335 | JPXX01.SZ0205 | Patient/stool | ST34 | M |
| S14161 | JPXX01.SZ0099 | Patient/stool | ST34 | M |
| S14097 | JPXX01.SZ0178 | Patient/stool | ST34 | M |
| S14242 | JPXX01.SZ0229 | Patient/stool | ST34 | W |
| S14023 | JPXX01.SZ0181 | Patient/stool | ST34 | M |
| S14203 | JPXX01.SZ0190 | Patient/stool | ST36 | W |
| S14339 | JPXX01.SZ0215 | Patient/stool | ST34 | S |
| S14173 | JPXX01.SZ0099 | Patient/stool | ST34 | W |
| S14346 | JPXX01.SZ0208 | Patient/stool | ST34 | S |
| S14133 | JPXX01.SZ0252 | Patient/stool | ST34 | W |
| S14194 | JPXX01.SZ0273 | Patient/stool | ST34 | W |
| S14207 | JPXX01.SZ0186 | Patient/stool | ST19 | N |
| S14045 | JPXX01.SZ0243 | Patient/stool | ST34 | W |
| S14100 | JPXX01.SZ0138 | Patient/stool | ST34 | M |
| S14126 | JPXX01.SZ0267 | Patient/stool | ST34 | N |
| S14058 | JPXX01.SZ0280 | Patient/stool | ST34 | S |
| S14264 | JPXX01.SZ0006 | Patient/stool | ST34 | S |
| S14391 | JPXX01.SZ0269 | Patient/stool | ST34 | S |
| S14205 | JPXX01.SZ0099 | Patient/stool | ST34 | W |
| S14340 | JPXX01.SZ0127 | Patient/stool | ST34 | W |
| S14135 | JPXX01.SZ0279 | Patient/stool | ST34 | S |
| S13007 | JPXX01.SZ0111 | Patient/stool | ST19 | N |
| S12190 | JPXX01.SZ0020 | Patient/stool | ST19 | W |
| S13274 | JPXX01.SZ0152 | Patient/stool | ST1544 | N |
| S13329 | JPXX01.SZ0145 | Patient/stool | ST19 | S |
| S13302 | JPXX01.SZ0092 | Patient/stool | ST34 | N |
| S13109 | JPXX01.SZ0277 | Patient/stool | ST19 | N |
| S13111 | JPXX01.SZ0154 | Patient/stool | ST19 | N |
| S13135 | JPXX01.SZ0015 | Patient/stool | ST1557 | N |
| S13110 | JPXX01.SZ0017 | Patient/stool | ST19 | N |
| S13072 | JPXX01.SZ0166 | Patient/stool | ST19 | N |
| S13287 | JPXX01.SZ0112 | Patient/stool | ST19 | N |
| S13180 | JPXX01.SZ0100 | Patient/stool | ST34 | S |
| S13068 | JPXX01.SZ0078 | Patient/stool | ST34 | W |
| S13280 | JPXX01.SZ0161 | Patient/stool | ST19 | N |
| S13273 | JPXX01.SZ0114 | Patient/stool | ST19 | W |
| S13147 | JPXX01.SZ0150 | Patient/stool | ST19 | W |
| S13358 | JPXX01.SZ0135 | Patient/stool | ST34 | W |
| S13293 | JPXX01.SZ0082 | Patient/stool | ST34 | S |
| S13348 | JPXX01.SZ0113 | Patient/stool | ST19 | W |
| S13336 | JPXX01.SZ0275 | Patient/stool | ST19 | S |
| S13279 | JPXX01.SZ0086 | Patient/stool | ST34 | S |
| S13209 | JPXX01.SZ0051 | Patient/stool | ST34 | S |
| S13292 | JPXX01.SZ0134 | Patient/stool | ST34 | S |
| S13097 | JPXX01.SZ0006 | Patient/stool | ST34 | S |
| S13131 | JPXX01.SZ0001 | Patient/stool | ST34 | S |
| S13183 | JPXX01.SZ0006 | Patient/stool | ST34 | S |
| S13008 | JPXX01.SZ0106 | Patient/stool | ST34 | S |
| S13004 | JPXX01.SZ0102 | Patient/stool | ST19 | M |
| S13118 | JPXX01.SZ0159 | Patient/stool | ST99 | W |
| S13107 | JPXX01.SZ0154 | Patient/stool | ST19 | W |
| S13108 | JPXX01.SZ0017 | Patient/stool | ST19 | W |
| S10085 | JPXX01.SZ0272 | Patient/stool | ST34 | S |
| S10134 | JPXX01.SZ0274 | Patient/stool | ST19 | W |
| S11003 | JPXX01.SZ0024 | Patient/stool | ST19 | W |
| S11075 | JPXX01.SZ0064 | Patient/stool | ST1544 | N |
| S11164 | JPXX01.SZ0017 | Patient/stool | ST19 | N |
| S11058 | JPXX01.SZ0036 | Patient/stool | ST19 | W |
| S11091 | JPXX01.SZ0069 | Patient/stool | ST34 | M |
| S12050 | JPXX01.SZ0054 | Patient/stool | ST34 | S |
| S12006 | JPXX01.SZ0016 | Patient/stool | ST19 | N |
| S10002 | JPXX01.SZ0120 | Patient/stool | ST19 | W |
| S10012 | JPXX01.SZ0007 | Patient/stool | ST34 | S |
| S10005 | JPXX01.SZ0019 | Patient/stool | ST19 | W |
| S11188 | JPXX01.SZ0023 | Patient/stool | ST19 | W |
| S11078 | JPXX01.SZ0062 | Patient/stool | ST34 | W |
| S11029 | JPXX01.SZ0053 | Patient/stool | ST34 | W |
| S11143 | JPXX01.SZ0105 | Patient/stool | ST34 | S |
| S11215 | JPXX01.SZ0047 | Patient/stool | ST34 | S |
| S11172 | JPXX01.SZ0048 | Patient/stool | ST34 | S |
| S11048 | JPXX01.SZ0052 | Patient/stool | ST34 | S |
| S11062 | JPXX01.SZ0126 | Patient/stool | ST34 | S |
| S12120 | JPXX01.SZ0052 | Patient/stool | ST34 | S |
| S12145 | JPXX01.SZ0091 | Patient/stool | ST34 | W |
| S12073 | JPXX01.SZ0030 | Patient/stool | ST34 | W |
| S12129 | JPXX01.SZ0052 | Patient/stool | ST34 | S |
| S12074 | JPXX01.SZ0111 | Patient/stool | ST34 | W |
| S10031 | JPXX01.SZ0125 | Patient/stool | ST34 | S |
| S10065 | JPXX01.SZ0003 | Patient/stool | ST34 | S |
| S10049 | JPXX01.SZ0004 | Patient/stool | ST34 | S |
| S11077 | JPXX01.SZ0066 | Patient/stool | ST34 | S |
| S11098 | JPXX01.SZ0067 | Patient/stool | ST34 | S |
| S11056 | JPXX01.SZ0005 | Patient/stool | ST34 | S |
| S11069 | JPXX01.SZ0083 | Patient/stool | ST34 | S |
| S11107 | JPXX01.SZ0004 | Patient/stool | ST34 | S |
| S11015 | JPXX01.SZ0043 | Patient/stool | ST34 | S |
| S11221 | JPXX01.SZ0060 | Patient/stool | ST19 | N |
| S11066 | JPXX01.SZ0049 | Patient/stool | ST34 | S |
| S11184 | JPXX01.SZ0054 | Patient/stool | ST34 | S |
| S11047 | JPXX01.SZ0089 | Patient/stool | ST34 | S |
| S11101 | JPXX01.SZ0068 | Patient/stool | ST19 | W |
| S11113 | JPXX01.SZ0042 | Patient/stool | ST34 | S |
| S11121 | JPXX01.SZ0078 | Patient/stool | ST34 | S |
| S11039 | JPXX01.SZ0013 | Patient/stool | ST34 | S |
| S11099 | JPXX01.SZ0077 | Patient/stool | ST34 | S |
| S11051 | JPXX01.SZ0120 | Patient/stool | ST34 | S |
| S11132 | JPXX01.SZ0080 | Patient/stool | ST34 | S |
| S12154 | JPXX01.SZ0086 | Patient/stool | ST34 | S |
| S12114 | JPXX01.SZ0032 | Patient/stool | ST34 | W |
| S12053 | JPXX01.SZ0008 | Patient/stool | ST19 | M |
| S12017 | JPXX01.SZ0068 | Patient/stool | ST19 | N |
| S12024 | JPXX01.SZ0041 | Patient/stool | ST1963 | S |
| S12054 | JPXX01.SZ0006 | Patient/stool | ST34 | S |
| S12028 | JPXX01.SZ0124 | Patient/stool | ST34 | S |
| S12078 | JPXX01.SZ0026 | Patient/stool | ST34 | W |
| S12019 | JPXX01.SZ0001 | Patient/stool | ST34 | S |
| S11063 | JPXX01.SZ0033 | Patient/stool | ST34 | M |
| S11211 | JPXX01.SZ0053 | Patient/stool | ST34 | M |
| S11251 | JPXX01.SZ0110 | Patient/stool | ST34 | W |
| S12045 | JPXX01.SZ0081 | Patient/stool | ST34 | M |
| S12065 | JPXX01.SZ0016 | Patient/stool | ST19 | M |
| S12079 | JPXX01.SZ0005 | Patient/stool | ST34 | S |
| S12103 | JPXX01.SZ0147 | Patient/stool | ST36 | W |
| S12157 | JPXX01.SZ0132 | Patient/stool | ST34 | S |
| S12168 | JPXX01.SZ0136 | Patient/stool | ST19 | M |
| S12187 | JPXX01.SZ0101 | Patient/stool | ST34 | S |
| S12203 | JPXX01.SZ0129 | Patient/stool | ST34 | M |
| S12213 | JPXX01.SZ0017 | Patient/stool | ST19 | W |
| S12240 | JPXX01.SZ0098 | Patient/stool | ST34 | S |
| S13076 | JPXX01.SZ0108 | Patient/stool | ST34 | M |
| S13124 | JPXX01.SZ0094 | Patient/stool | ST34 | S |
| S13168 | JPXX01.SZ0076 | Patient/stool | ST19 | W |
| S13263 | JPXX01.SZ0130 | Patient/stool | ST34 | S |
| S13285 | JPXX01.SZ0164 | Patient/stool | ST34 | M |
| S13290 | JPXX01.SZ0139 | Patient/stool | ST34 | S |
| S13291 | JPXX01.SZ0142 | Patient/stool | ST34 | M |
| S14008 | JPXX01.SZ0173 | Patient/stool | ST19 | W |
| S14009 | JPXX01.SZ0180 | Patient/stool | ST34 | W |
| S14025 | JPXX01.SZ0177 | Patient/stool | ST34 | M |
| S14028 | JPXX01.SZ0174 | Patient/stool | ST19 | S |
| S14034 | JPXX01.SZ0062 | Patient/stool | ST34 | W |
| S14037 | JPXX01.SZ0086 | Patient/stool | ST34 | M |
| S14050 | JPXX01.SZ0195 | Patient/stool | ST19 | N |
| S14051 | JPXX01.SZ0196 | Patient/stool | ST34 | M |
| S14053 | JPXX01.SZ0183 | Patient/stool | ST34 | N |
| S14108 | JPXX01.SZ0238 | Patient/stool | ST19 | N |
| S14128 | JPXX01.SZ0242 | Patient/stool | ST34 | W |
| S14206 | JPXX01.SZ0188 | Patient/stool | ST34 | M |
| S14208 | JPXX01.SZ0218 | Patient/stool | ST34 | S |
| S14213 | JPXX01.SZ0235 | Patient/stool | ST36 | N |
| S14222 | JPXX01.SZ0179 | Patient/stool | ST34 | N |
| S14237 | JPXX01.SZ0198 | Patient/stool | ST34 | W |
| S14239 | JPXX01.SZ0240 | Patient/stool | ST34 | W |
| S14256 | JPXX01.SZ0192 | Patient/stool | ST34 | W |
| S14261 | JPXX01.SZ0224 | Patient/stool | ST34 | S |
| S14267 | JPXX01.SZ0187 | Patient/stool | ST19 | N |
| S14297 | JPXX01.SZ0201 | Patient/stool | ST34 | S |
| S14312 | JPXX01.SZ0207 | Patient/stool | ST34 | M |
| S14329 | JPXX01.SZ0217 | Patient/stool | ST34 | M |
| S14342 | JPXX01.SZ0206 | Patient/stool | ST34 | M |
| S14344 | JPXX01.SZ0202 | Patient/stool | ST34 | W |
| S14357 | JPXX01.SZ0230 | Patient/stool | ST34 | M |
| S14360 | JPXX01.SZ0233 | Patient/stool | ST19 | M |
| S14361 | JPXX01.SZ0197 | Patient/stool | ST34 | M |
| S14373 | JPXX01.SZ0234 | Patient/stool | ST19 | M |
| S14382 | JPXX01.SZ0214 | Patient/stool | ST34 | M |
| S14388 | JPXX01.SZ0138 | Patient/stool | ST34 | W |
| S14396 | JPXX01.SZ0257 | Patient/stool | ST34 | N |
| S14403 | JPXX01.SZ0236 | Patient/stool | ST36 | W |
| S14406 | JPXX01.SZ0265 | Patient/stool | ST19 | N |
| S14433 | JPXX01.SZ0251 | Patient/stool | ST34 | N |
| SM120011 | JPXX01.SZ0320 | Ready-to-eat/meat | ST516 | N |
| SM120021 | JPXX01.SZ0321 | Livestock/meat | ST34 | M |
| SM120031 | JPXX01.SZ0005 | Ready-to-eat/flour products | ST34 | S |
| SM110004 | JPXX01.SZ0153 | Poultry/meat | ST19 | N |
| SM110006 | JPXX01.SZ0112 | Livestock/meat | ST19 | N |
| SM110007 | JPXX01.SZ0153 | Poultry/meat | ST19 | N |
| SM110008 | JPXX01.SZ0153 | Ready-to-eat/flour products | ST19 | N |
| SM110011 | JPXX01.SZ0090 | Livestock/meat | ST34 | S |
| SM110018 | JPXX01.SZ0001 | Aquatic product/paludina | ST34 | M |
| SM110019 | JPXX01.SZ0104 | Aquatic product/fish | ST34 | S |
| SM110010 | JPXX01.SZ0153 | Poultry/meat | ST19 | N |
| SM130013 | JPXX01.SZ0138 | Livestock/meat | ST34 | N |
| SM130017 | JPXX01.SZ0163 | Poultry/meat | ST241 | N |
| SM130034 | JPXX01.SZ0034 | Poultry/meat | ST19 | N |
| SM130044 | JPXX01.SZ0039 | Livestock/meat | ST36 | N |
| SM130047 | JPXX01.SZ0039 | Livestock/meat | ST36 | N |
| SM130049 | JPXX01.SZ0031 | Livestock/meat | ST34 | N |
| SM130052 | JPXX01.SZ0031 | Livestock/meat | ST34 | N |
| SM140003 | JPXX01.SZ0145 | Poultry/meat | ST19 | N |
| SM140004 | JPXX01.SZ0035 | Livestock/meat | ST34 | M |
| SM140009 | JPXX01.SZ0119 | Livestock/meat | ST34 | S |
| SM140010 | JPXX01.SZ0119 | Poultry/meat | ST34 | S |
| SM140014 | JPXX01.SZ0057 | Livestock/meat | ST34 | M |
| SM140020 | JPXX01.SZ0075 | Livestock/meat | ST34 | N |
| SM140022 | JPXX01.SZ0137 | Livestock/meat | ST19 | N |
| SM140023 | JPXX01.SZ0034 | Poultry/meat | ST19 | N |
| SM140024 | JPXX01.SZ0034 | Poultry/meat | ST19 | N |
| SM140034 | JPXX01.SZ0070 | Livestock/meat | ST34 | M |
| SM140039 | JPXX01.SZ0154 | Poultry/meat | ST19 | N |
| SM140046 | JPXX01.SZ0002 | Poultry/meat | ST34 | S |
| SM140056 | JPXX01.SZ0178 | Livestock/meat | ST34 | N |
| SM140073 | JPXX01.SZ0232 | Livestock/meat | ST34 | N |
| SM140089 | JPXX01.SZ0216 | Livestock/meat | ST34 | S |

^a^ Different categories of biofilm production are represented by the following abbreviations: N, non-biofilm producer; W, weak biofilm producer; M, moderate biofilm producer; S, strong biofilm producer.

TABLE S2 Occurrence of antimicrobial resistance in *Salmonella* Typhimurium isolates recovered from food.

| Year | Percentage (%) of non-susceptible isolates | | | | | | | | | | | | | | | | | |
| --- | --- | --- | --- | --- | --- | --- | --- | --- | --- | --- | --- | --- | --- | --- | --- | --- | --- | --- |
|  | AMP | AMC | CEP | CAZ | CRO | FEP | FOX | AMK | GEN | KAN | STR | NAL | CIP | LVX | SXT | TMP | CHL | TCY |
| 2014 (n=15) | 53.3 | 20 | 0 | 0 | 0 | 0 | 0 | 0 | 33.3 | 46.7 | 80 | 73.3 | 20 | 0 | 53.3 | 53.3 | 60 | 66.7 |
| 2013 (n=7) | 57.1 | 28.6 | 0 | 0 | 0 | 0 | 0 | 57.1 | 71.4 | 71.4 | 85.7 | 85.7 | 57.1 | 57.1 | 0 | 0 | 42.9 | 85.7 |
| 2012 (n=3) | 66.7 | 33.3 | 0 | 0 | 0 | 0 | 0 | 0 | 33.3 | 33.3 | 66.7 | 100 | 0 | 0 | 0 | 0 | 33.3 | 66.7 |
| 2011 (n=8) | 25 | 0 | 0 | 0 | 0 | 0 | 0 | 0 | 25 | 25 | 62.5 | 100 | 12.5 | 0 | 12.5 | 12.5 | 50 | 37.5 |

AMP, ampicillin; AMC, amoxicillin; CEP, cephalothin; CAZ, ceftazidime; CRO, ceftriaxone; FEP, cefepime; FOX, cefoxitin; AMK, amikacin; GEN, gentamicin; KAN, kanamycin; STR, streptomycin; NAL, nalidixic acid; CIP, ciprofloxacin; LVX, levofloxacin; SXT, sulfamethoxazole; TMP, trimethoprim; CHL, chloramphenicol; TCY, tetracycline.

TABLE S3 Occurrence of resistance to a particular antimicrobial by year among *Salmonella* Typhimurium clinical isolates

| Year | Percentage (%) of non-susceptible isolates | | | | | | | | | | | | | | | | | |
| --- | --- | --- | --- | --- | --- | --- | --- | --- | --- | --- | --- | --- | --- | --- | --- | --- | --- | --- |
|  | AMP | AMC | CEP | CAZ | CRO | FEP | FOX | AMK | GEN | KAN | STR | NAL | CIP | LVX | SXT | TMP | CHL | TCY |
| 2014 | 76.5 | 47.9 | 35.3 | 23.5 | 21.0 | 14.3 | 1.7 | 7.6 | 31.9 | 37.0 | 95.0 | 69.7 | 87.4 | 1.7 | 37.8 | 38.7 | 45.4 | 83.2 |
| 2013 | 67.4 | 46.7 | 9.8 | 8.7 | 7.6 | 5.4 | 1.1 | 12.0 | 30.4 | 39.1 | 93.5 | 58.7 | 71.7 | 0 | 42.4 | 43.5 | 47.8 | 70.7 |
| 2012 | 71.4 | 50.8 | 4.8 | 3.2 | 1.6 | 1.6 | 0 | 1.6 | 44.4 | 50.8 | 87.3 | 73.0 | 85.7 | 1.6 | 54.0 | 52.4 | 54.0 | 74.6 |
| 2011 | 71.1 | 53.9 | 21.1 | 9.2 | 11.8 | 6.6 | 1.3 | 11.8 | 35.5 | 53.9 | 88.2 | 69.7 | 93.4 | 9.2 | 38.2 | 39.5 | 55.3 | 69.7 |
| 2010 | 79.1 | 55.8 | 18.6 | 2.3 | 0 | 0 | 4.7 | 7.0 | 44.2 | 60.5 | 62.8 | 93.0 | 95.3 | 4.7 | 60.5 | 51.2 | 69.8 | 79.1 |

AMP, ampicillin; AMC, amoxicillin; CEP, cephalothin; CAZ, ceftazidime; CRO, ceftriaxone; FEP, cefepime; FOX, cefoxitin; AMK, amikacin; GEN, gentamicin; KAN, kanamycin; STR, streptomycin; NAL, nalidixic acid; CIP, ciprofloxacin; LVX, levofloxacin; SXT, sulfamethoxazole; TMP, trimethoprim; CHL, chloramphenicol; TCY, tetracycline.
